# Local anaesthesia decreases nerve growth factor induced masseter hyperalgesia

**DOI:** 10.1038/s41598-020-71620-8

**Published:** 2020-09-22

**Authors:** Yuri M. Costa, Fernando G. Exposto, Eduardo E. Castrillon, Paulo César R. Conti, Leonardo R. Bonjardim, Peter Svensson

**Affiliations:** 1 Department of Biosciences, Piracicaba Dental School, University of Campinas, Av. Limeira, 901, Piracicaba, CEP 13414-903 Brazil; 2 Section of Orofacial Pain and Jaw Function, Department of Dentistry and Oral Health, Aarhus University, Aarhus, Denmark; 3 Scandinavian Center for Orofacial Neurosciences (SCON), Aarhus, Denmark; 4 Department of Prosthodontics, Bauru School of Dentistry, University of São Paulo, Bauru, Brazil; 5 Section of Head and Face Physiology, Department of Biological Sciences, Bauru School of Dentistry, University of São Paulo, Bauru, Brazil; 6 Faculty of Odontology, Malmö University, Malmö, Sweden

**Keywords:** Headache, Experimental models of disease

## Abstract

The aim of this investigation was to evaluate the effects of local anaesthesia on nerve growth factor (NGF) induced masseter hyperalgesia. Healthy participants randomly received an injection into the right masseter muscle of either isotonic saline (IS) given as a single injection (n = 15) or an injection of NGF (n = 30) followed by a second injection of lidocaine (NGF + lidocaine; n = 15) or IS (NGF + IS; n = 15) in the same muscle 48 h later. Mechanical sensitivity scores of the right and left masseter, referred sensations and jaw pain intensity and jaw function were assessed at baseline, 48 h after the first injection, 5 min after the second injection and 72 h after the first injection. NGF caused significant jaw pain evoked by chewing at 48 and 72 h after the first injection when compared to the IS group, but without significant differences between the NGF + lidocaine and NGF + IS groups. However, the mechanical sensitivity of the right masseter 5 min after the second injection in the NGF + lidocaine group was significantly lower than the second injection in the NGF + IS and was similar to the IS group. There were no significant differences for the referred sensations. Local anaesthetics may provide relevant information regarding the contribution of peripheral mechanisms in the maintenance of persistent musculoskeletal pain.

## Introduction

Intramuscular injection of nerve growth factor (NGF) has been successfully applied to evoke long-lasting mechanical sensitization of the skeletal muscles^1–4^. In particular, NGF-induced masseter hyperalgesia can last for up to 2 weeks and is associated with reduced pressure pain thresholds (PPT) and pain evoked by jaw function^5^. This distinctive feature of sustained muscle soreness in association with the possibility of establishing cause-effect relationships has made the NGF pain model well suited to investigate the clinical presentation and possibly the underlying mechanisms of myofascial pain. Indeed, human experimental muscle pain models are regarded as the bridge between basic and clinical pain research, and, therefore, can help in translating mechanistic knowledge into clinical practice^6–8^.

The first report that systematically addressed the sensitivity changes following intramuscular NGF into the jaw muscles highlighted that it was uncertain if central mechanisms would be the primary contributor of the observed delayed and long-term (up to 7 days) muscle hyperalgesia following a single injection of NGF^5^. Although it is reasonably well established that both peripheral and central sensitization mechanisms underlie the hyperalgesic effects of the NGF^4,9–13^, it is still not yet fully established how they interact to initiate and, especially, to maintain these sensory outcomes.

Local anaesthetics have been applied to investigate the role of peripheral inputs in both, experimental pain models and clinical pain conditions^14–18^ and it is logical to presume hypoalgesia/hypoesthesia following the administration of local anaesthetics. The rationale is that if peripheral mechanisms considerably account for the sensory signs and symptoms, a successful block of the peripheral tissue would likely attenuate, at least to a reasonable extent, the observed increased pain sensitivity^17,18^. Previous investigations did not observe such expected decrease in NGF-sensitized muscles after intramuscular injection of ropivacaine 0.25%^19^. In addition, a recent network meta-analysis showed that the effect of intramuscular local anaesthesia on pain reduction and muscle sensitivity in patients with masticatory myofascial pain is of minor clinical relevance^20^. Overall, these findings could suggest either that the effects of intramuscular administration of anaesthetics are small or that central changes may have hindered the peripheral anaesthetic block. The latter has been particularly the focus of previous investigations and it has also been argued that the NGF-induced muscle sensitization is partly due to central changes^2,3,5,9,19,21,22^. In addition, the effect of local anaesthesia is possibly more challenging to be detected in pain conditions where both mechanisms are present, which calls for further investigations.

It is also important to highlight that local anaesthesia alone cannot rule out the presence of central sensitization but it could rather serve to underpin the contribution of peripheral mechanisms in painful conditions where central and peripheral sensitization are likely present, for instance, NGF-induced muscle hyperalgesia^9,12^. Therefore, local anaesthesia of NGF-sensitized muscles could contribute to further elucidate the mechanisms of long-lasting masseter hyperalgesia, which could shed light on the pathophysiology of the myogenous types of temporomandibular disorders (TMD), given the abovementioned translational aspects of the NGF pain model.

That being said, the primary aim of this investigation was to evaluate the effects of local anaesthesia applied to the muscle on the NGF-induced masseter hyperalgesia. Considering previous evidence that peripheral block seems not to reduce the sensitivity following NGF injection in limb muscles^19^, we hypothesized a priori that lidocaine injections would not substantially decrease the masseter mechanical sensitivity induced by intramuscular administration of NGF.

## Results

### Mechanical sensitivity and entropy

Forty-five healthy participants were equally divided and included in the IS, NGF + lidocaine and NGF + IS groups with, respectively, mean age (SD) of 25.4 (3.6) and 47% women, mean age (SD) of 25.8 (10.2) and 60% women, and mean age (SD) of 25.3 (4.8) and 47% women. There were no significant between-group differences in the sex distribution (*p* = 0.700) and age (*p* = 0.985).

**Figure 1:**
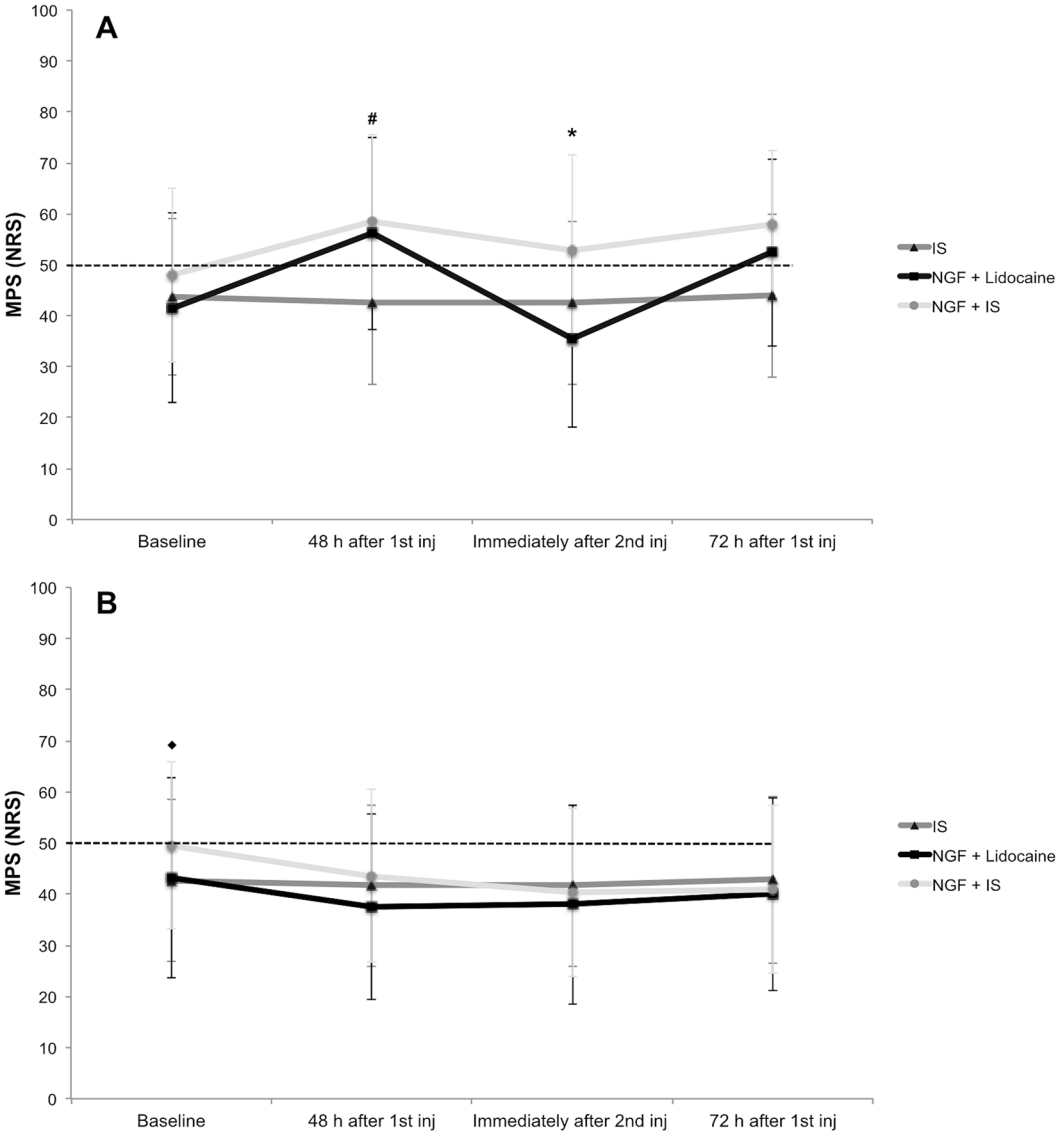
Mechanical pain sensitivity (MPS) scores, i.e., average of 1 and 2 kg forces, from the injected (right side—**A**) and control (left side—**B**) masseter before the injection (baseline), 48 h after the first injection, 5 min after the second injection and 72 h after the first injection of either isotonic saline (IS; n = 15) or nerve growth factor (1st injection) and lidocaine (2nd injection) (NGF + lidocaine; n = 15) or NGF (1st injection) and IS (2nd injection) (NGF + IS; n = 15). NRS = 0–50–100, numeric rating scale. The dashed lines indicate the pain threshold. **#** = Significant within-group differences when compared to baseline values for the NGF + lidocaine (*p* < 0.001) and NGF + IS (*p* < 0.001). * = Significant between-group differences for the NGF + lidocaine when compared to NGF + IS group (*p* = 0.005). ◆ = significant within-group differences when compared to 5 min after the second injection (*p* < 0.001) and 72 h after the first injection values (*p* < 0.003) for the NGF + IS. There were neither baseline differences among the groups nor within-group differences for the IS group (*p* > 0.050). Error-bars indicate the standard deviation.

There was a significant interaction between group and session for the mechanical sensitivity scores of the right masseter (injected side), F_6, 126_ = 8.48, *p* < 0.001 and partial η^2^ = 0.28, where the mechanical sensitivity 5 min after the second injection in the NGF + lidocaine group was lower than the second injection in the NGF + IS (Tukey: *p* = 0.005) and was similar to the IS group (Tukey: *p* = 0.870) (Fig. 1A). In addition, there was a significant main effect of group, session and force for the mechanical sensitivity scores of the right masseter (injected side), respectively, F_2, 42_ = 4.89, *p* = 0.012 and partial η^2^ = 0.18, F_3, 126_ = 20.27, *p* < 0.001 and partial η^2^ = 0.32, and F_1, 42_ = 225.60, *p* < 0.001 and partial η^2^ = 0.84. The NGF + IS group presented greater mechanical sensitivity when compared to the IS group (Tukey: *p* = 0.011), the mechanical sensitivity scores 48 and 72 h after the first injection where higher than baseline and 5 min after the second injection values (Tukey: *p* < 0.001), and the mechanical sensitivity scores with 2 kg were higher than 1 kg force (Tukey: *p* < 0.001).

The left masseter (control side) presented significant interactions between group and session for the mechanical sensitivity scores, F_6, 126_ = 2.17, *p* = 0.049 and partial η^2^ = 0.09, where the scores at baseline where higher than 5 min after the second injection (Tukey: *p* = 0.001) and 72 h after the first injection (Tukey: *p* = 0.003) for the NGF + IS group (Fig. 1B). Finally, there were main effects of session and force for the mechanical sensitivity scores of the left masseter (control side), respectively, F_3, 126_ = 7.53, *p* < 0.001 and partial η^2^ = 0.15, and F_1, 42_ = 104.01, *p* < 0.001 and partial η^2^ = 0.71. The greatest mechanical sensitivity was presented at baseline session (Tukey: *p* < 0.010) and with 2 kg (Tukey: *p* < 0.001).

**Figure 2:**
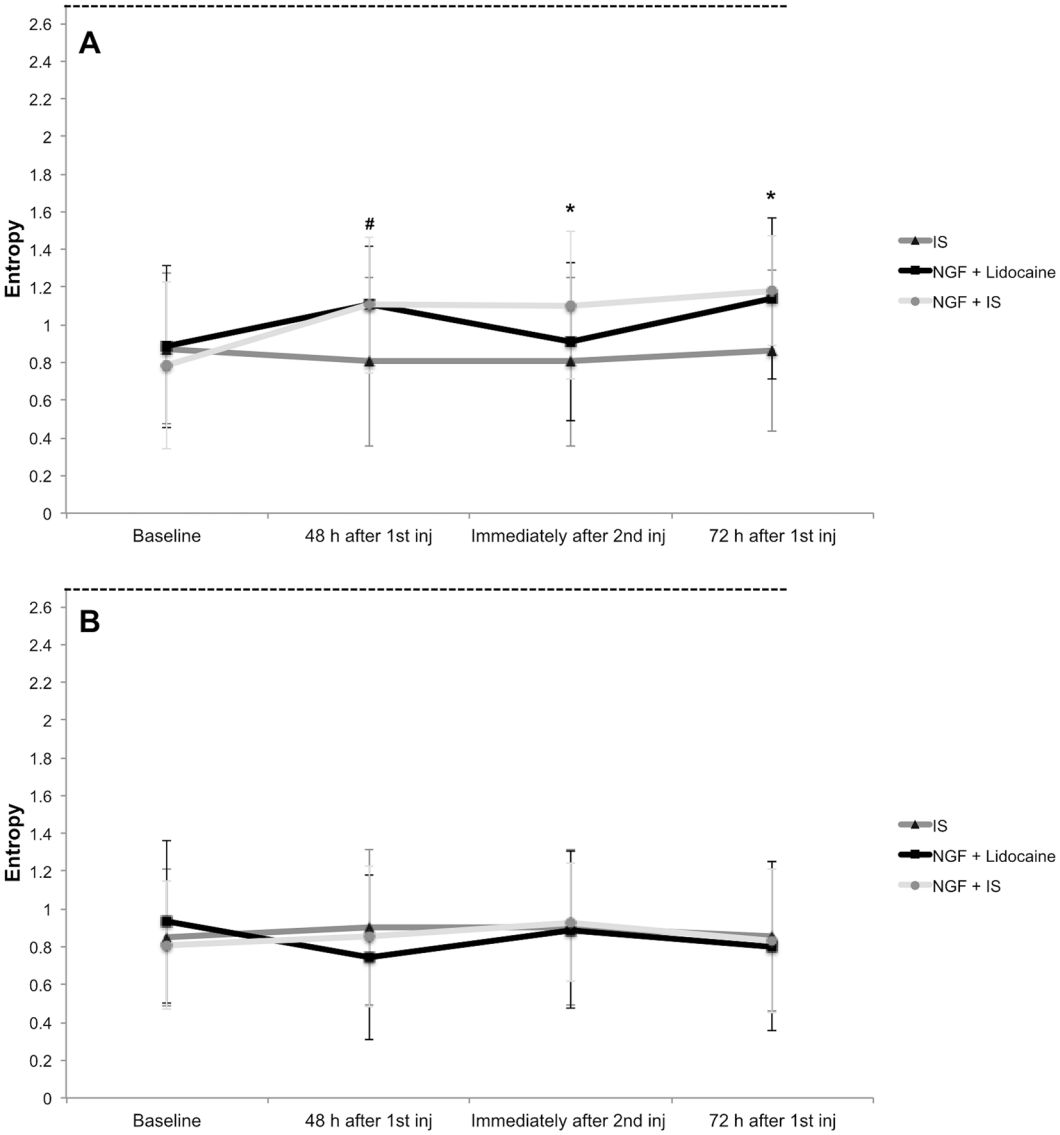
Entropy scores, i.e., average of 1 and 2 kg forces, from the injected (right side—**A**) and control (left side—**B**) masseter before the injection (baseline), 48 h after the first injection, 5 min after the second injection and 72 h after the first injection of either isotonic saline (IS; n = 15) or nerve growth factor (1st injection) and lidocaine (2nd injection) (NGF + lidocaine; n = 15) or NGF (1st injection) and IS (2nd injection) (NGF + IS; n = 15). The dashed lines indicate maximum entropy. # = Significant within-group differences when compared to baseline values for the NGF + lidocaine (*p* = 0.046) and NGF + IS (*p* < 0.001). * = Significant within-group differences when compared to baseline for the NGF + IS group (*p* < 0.001). There were neither baseline differences among the groups nor within-group differences for the IS group (*p* > 0.050). Error-bars indicate the standard deviation.

There was a significant interaction between group and session for the entropy scores of the right masseter (injected side), F_6, 126_ = 4.94, *p* < 0.001 and partial η^2^ = 0.19, where the entropy was increased at 48 h after the first injection when compared to baseline values in the NGF + lidocaine group (Tukey: *p* = 0.046), and was increased at 48 h after the first injection, 5 min after the second injection and 72 h after the first injection when compared to baseline in the NGF + IS group (Fig. 2A). Furthermore, the entropy scores of the right masseter (injected site) showed significant main effect of session (F_3, 126_ = 8.35, *p* < 0.001 and partial η^2^ = 0.16), where the values at 48 and 72 h after the first injection were higher than baseline (Tukey: *p* < 0.001). Finally, the entropy scores of the left side did not present significant main effects (*p* > 0.050), and although there was a significant interaction between force and group (F_2, 42_ = 3.53, *p* = 0.038 and partial η^2^ = 0.14), the multiple comparison post-hoc analyses were non-significant (*p* > 0.050) (Fig. 2B)^20^.

### Referred sensation

Table 1 shows the distribution of referred sensation upon pressure with either 1 or 2 kg force for the right (injected side) and left (control side) masseter within each group considering the different assessment time points. There were neither significant between-group (*p* > 0.012) nor within-group differences (*p* > 0.016) for the presence of referred sensation considering the *p* values adjusted for multiple comparisons (Table 1).

**Table 1:** Distribution of referred sensation upon pressure application of either 1 or 2 kg before the injection (baseline), 48 h after the first injection, 5 min after the second injection and 72 h after the first injection of either isotonic saline (IS) or nerve growth factor (1st injection) and lidocaine (2nd injection) (NGF + lidocaine) or NGF (1st injection) and IS (2nd injection) (NGF + IS).

Session	Injected side (right)	Control side (left)
**IS (n = 15)**		
Baseline	5 (33%)	5 (33%)
48 h after 1st injection	4 (29%)	5 (33%)
72 h after 1st injection	4 (27%)	6 (40%)
**NGF + lidocaine (n = 15)**		
Baseline	5 (33%)	5 (33%)
48 h after 1st injection	3 (20%)	3 (20%)
5 min after 2nd injection	1 (6%)	1 (6%)
72 h after 1st injection	5 (33%)	2 (13%)
**NGF + IS (n = 15)**		
Baseline	6 (40%)	8 (53%)
48 h after 1st injection	8 (53%)	6 (46%)
5 min after 2nd injection	5 (33%)	6 (40%)
72 h after 1st injection	7 (46%)	5 (33%)

There were neither significant between-group (*p* > 0.012) nor within-group differences (*p* > 0.016) for the presence of referred sensation considering the *p* values adjusted for multiple comparisons after Bonferroni correction.

### Jaw pain and function

**Figure 3:**
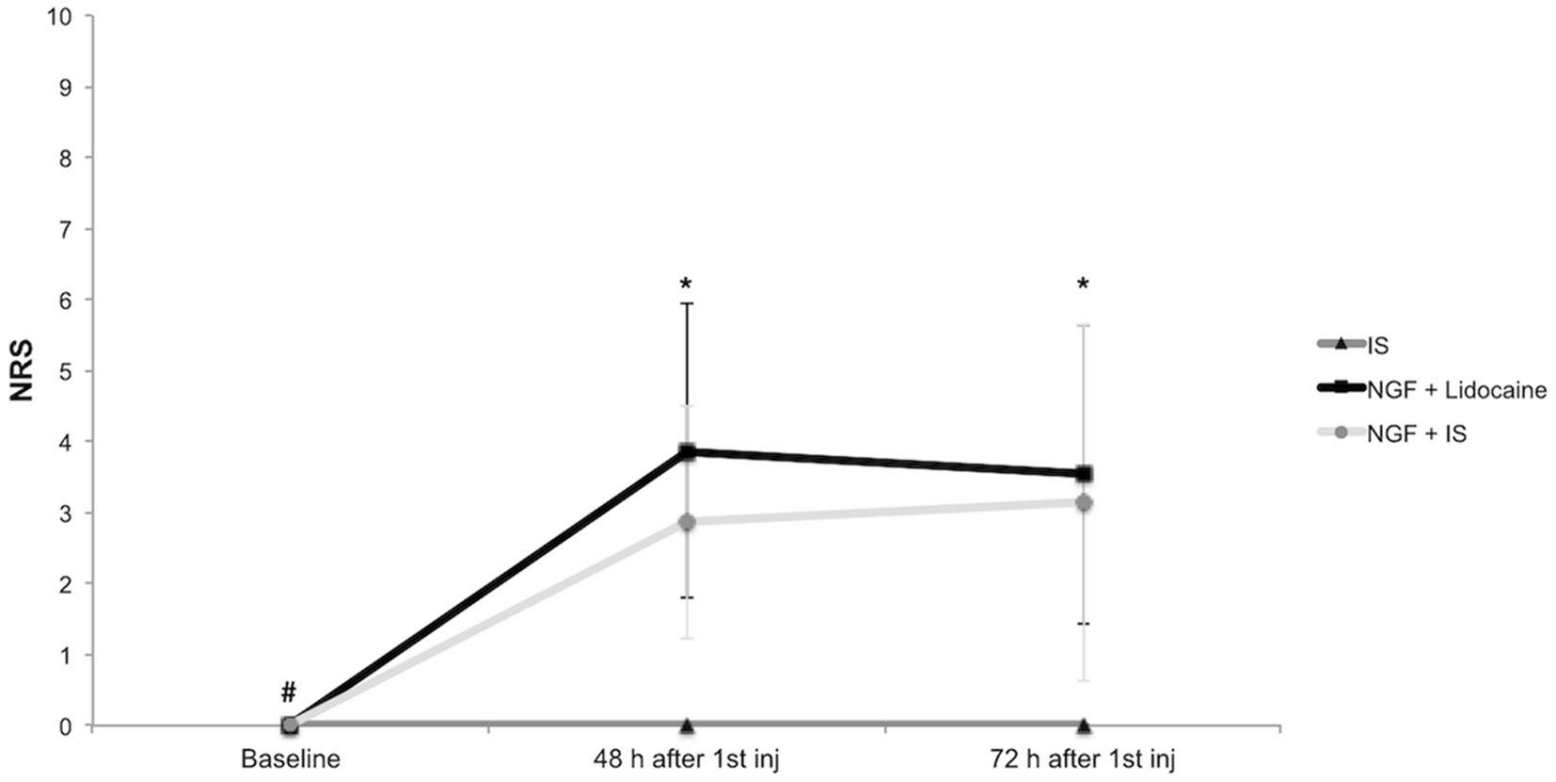
Jaw pain intensity evoked by chewing before the injection (baseline), 48 and 72 h after the first injection of either isotonic saline (IS; n = 15) or nerve growth factor (1st injection) and lidocaine (2nd injection) (NGF + lidocaine; n = 15) or NGF (1st injection) and IS (2nd injection) (NGF + IS; n = 15). NRS = 0–10, numeric rating scale. **#** = Significant within-group differences when compared to 48 and 72 h after the first injection for the NGF + lidocaine and NGF + IS group (*p* < 0.001). * = Significant between-group differences for the NGF + lidocaine and NGF + IS when compared to IS group (*p* < 0.001). There were neither baseline differences among the groups, nor between-group differences for the NGF + lidocaine and NGF + IS groups and within-group differences for the IS group (*p* > 0.050). Error-bars indicate the standard deviation.

The intramuscular administration of NGF caused significant jaw pain evoked by chewing at 48 and 72 h after the first injection when compared to the IS group and to the baseline values (Tukey: *p* < 0.050) (Fig. 3). However, there were no differences between NGF + lidocaine group and NGF + IS groups (Tukey: *p* > 0.050) (Fig. 3). No significant effect of NGF on jaw pain at rest was observed and IS injections did not cause any significant jaw pain (Tukey: *p* > 0.050).

The jaw function was also negatively affected in the NGF + lidocaine and NGF + IS groups (Table 2). Self- reported chewing ability and JFLS global scores were higher at 48 and 72 h after the first injection when compared to the IS group (between-group difference) and to baseline values (within-group differences) (Tukey: *p* < 0.050).

**Table 2:** Self-reported chewing ability and global score of the jaw function limitation scale (JFLS) before the injection (baseline), 48 h after the first injection and 72 h after the first injection of either isotonic saline (IS) or nerve growth factor (1st injection) and lidocaine (2nd injection) (NGF + lidocaine) or NGF (1st injection) and IS (2nd injection) (NGF + IS).

Session	Chewing ability	JFLS
**IS (n = 15)**		
Baseline	3.0 (1.0)^Aa^	0.0 (0.1)^Aa^
48 h after 1st injection	3.0 (1.0)^Aa^	0.0 (0.1)^Aa^
72 h after 1st injection	3.0 (0.0)^Aa^	0.0 (0.0)^Aa^
**NGF + lidocaine (n = 15)**		
Baseline	3.0 (0.0)^Aa^	0.0 (0.1)^Aa^
48 h after 1st injection	10.0 (7.0)^Bb^	1.4 (1.8)^Bb^
72 h after 1st injection	12.0 (9.0)^Bb^	1.1 (1.5)^Bb^
**NGF + IS (n = 15)**		
Baseline	3.0 (0.0)^Aa^	0.0 (0.2)^Aa^
48 h after 1st injection	12.0 (8.0)^Bb^	1.3 (1.3)^Bb^
72 h after 1st injection	11.2 (4.9)^Bb^	0.9 (1.0)^Bb^

Different lowercase letters under the same group heading indicate significant within-group differences following pairwise post hoc comparisons (*p* < 0.050). Different uppercase letters in the same session headings indicate significant between-group differences following pairwise post-hoc comparisons (*p* < 0.050).

## Discussion

This randomized, double blind placebo-controlled trial aimed to assess the analgesic effects of intramuscular lidocaine in NGF-sensitized masseter muscles, which can provide relevant insight regarding the sensitization mechanisms of intramuscular administration of NGF. The main findings can be summarized as follows: (a) local anaesthesia of the masseter muscle can reduce NGF-induced hyperalgesia; (b) the anaesthetic effect is short-lived and does not significantly interfere with the report of referred sensation.

This is the first report of decreased sensitivity to pressure stimuli following intramuscular anaesthetic injections into experimentally sensitized masseter muscles, which suggests that peripheral mechanisms are important contributors to the delayed NGF-induced mechanical hyperalgesia. Evidence from in vitro and animal studies has elucidated the effects of NGF on nociceptive processing, which include, but are not limited to, (1) activation of the tyrosine kinase A (TrkA) receptor that increases the phosphorylation and insertion of the ion channel receptor transient receptor potential vanilloid 1 (TRPV1), which, in turn, leads to peripheral sensitization of high threshold afferents, presumably nociceptive fibers^9,10,12,23^; (2) antidromic transport of NGF through the endocytosis of the ligand-receptor complex (NGF-TrkA), which is able to change the expression of neuropeptides and sodium channels that are associated with increased excitability within the central nervous system (CNS) and further expression of TRPV1 in the peripheral terminal^24–26^. Nonetheless, it has to be acknowledged that the abovementioned evidence is mostly related to NGF-induced thermal sensitization and it is still unclear if these same mechanisms are also involved in NGF-induced mechanical sensitization.

Interestingly, mechanical sensitization following systemic administration of NGF in rats occurs later than thermal sensitization and it is has been suggested that central mechanisms also account for NGF induced mechanical hyperalgesia^22,27,28^. In addition, the well documented delayed onset and long-lasting muscle hyperalgesia in humans following a single dose of intramuscular injected NGF is usually associated with central sensitization mechanisms^1,3,5,19^. Although we do not have a detailed time course evaluation of the hyperalgesic effects, our findings of jaw pain evoked by chewing and decreased self-reported jaw function 48 h after NGF injection are in accordance with previous evidence that systematically addressed the duration of mechanical sensitivity changes following intramuscular administration of NGF into the masseter muscle^2,3,5^. Therefore, peripheral and central sensitization are likely to be involved in the masseter hyperalgesia that was present 48 h after the injection of NGF. Nonetheless, our results suggest that even if central mechanisms are most likely associated with the long-lasting sensitization effects of the NGF, peripheral inputs seem to play a relevant role in the maintenance of this delayed hyperalgesic state, at least at the injection site and neighboring region, considering that local anaesthesia decreased the mechanical sensitivity, while the IS did not cause any significant change. Therefore, this finding indicates that that peripheral mechanisms may indeed be important contributors to muscle hyperalgesia, which has important diagnostic and management implications. Of course, much still needs to be done in order to elucidate all the possible mechanisms, but this particular investigation can contribute to further understanding of muscle pain.

A previous similar study failed to demonstrate an analgesic effect following intramuscular anaesthesia of NGF-sensitized supraspinatus muscles, and central sensitization mechanisms were regarded as the most likely explanation^19^. It was argued that the nociceptive barrage as a consequence of the mechanical trauma of the injection itself was amplified due to the ongoing central sensitization, which dimmed the effect of the anaesthetic block^19^. It was then concluded that the clinical usefulness of local anaesthesia to distinguish the muscle as primary source of pain when central sensitization is present is very limited^19^. Nevertheless, our results suggest the opposite, considering that we were able to identity a significant analgesic effect 5 min after the local anaesthesia with lidocaine, 48 h after the injection of NGF. There are methodological differences that possibly account for these contradictory findings, e.g., the ratio between the dose of anaesthetic solution and muscle volume that was applied to evaluate the analgesic effect^19^. There are no reports where the structural assessment of both muscles, i.e., masseter and supraspinatus, has been performed, which precludes direct comparisons. Nevertheless, the cross-sectional area and thickness of the masseter muscle at rest measured by ultrasonography have been reported to range, respectively, from 3.0 to 16.1 mm^2^ and from 8.7 to 11.5 mm^29–31^. On the other hand, cross-sectional area and thickness of the supraspinatus muscle at rest can range, respectively, from 48 to 77 mm^2^ and from 17 to 23 mm^32,33^, which clearly indicates a substantially lower volume of the masseter muscle in comparison with the supraspinatus. Therefore, the anaesthetic solution volume of 6–10 mL may not have been sufficient to cause a significant analgesic effect in the NGF-sensitized supraspinatus^19^ like the one seen in the present study for the masseter muscle. In addition, the supraspinatus muscle lies beneath the trapezius, which should be taken into account when interpreting its mechanical sensitivity to pressure stimuli. In addition, the small sample size and the crossover design without clear information on how possible carry-over effects were taken into consideration may have made the inhibitory effects of the local anaesthesia indiscernible^19^.

The non-injected side did not present a significant modulation of mechanical sensitivity 5 min after the lidocaine injection, which was an expected finding considering the mechanisms of action of local anaesthesia. Nevertheless, the observed slight decrease in mechanical sensitivity of the left masseter following the second injection in the NGF + IS group might be the result of the activation of descending inhibitory pathways due to the additional trauma of the injection in the already sensitized right masseter. Contralateral sensory loss has also been observed following ipsilateral nociceptive activity evoked by capsaicin^34^. The same tendency was also observed for the NGF + lidocaine group, although the difference was not significant. It is likely that the lidocaine might have counterbalanced the concomitant nociceptive barrage evoked by the mechanical trauma of the injection, thus, reducing the magnitude of the inhibitory pain modulation.

The secondary findings also provided interesting results, e.g., the entropy scores 5 min after the lidocaine injection of NGF sensitized masseter returned to levels similar to baseline, whereas remained increased following the IS injection in the NGF + IS group, although there were no between-group differences (Fig. 2A). Overall, the entropy scores were low, which corroborates previous findings of similar distribution of the mechanical sensitivity within the masseter, i.e., low variability among the assessment sites^35,36^. Likewise, the increased entropy present 48 h after NGF injection was also an expected finding, considering previous evidence from experimental muscle pain investigations^2,35^. The increased entropy indicates a more diverse distribution of mechanical sensitivity scores within the assessed muscles. However, for the first time, it was observed that a lidocaine injection was able to not only reduce mechanical hyperalgesia, but also to decrease the heterogeneity of mechanical sensitivity. Taken together, the low and more uniform sensory scores following local anaesthesia may suggest that the dose was adequate to block the nociceptive signalling within the whole masseter muscle.

The report of referred sensation was not significantly changed following either NGF or lidocaine injections, which indicates that sensitization mechanisms might not be the main cause of referred sensations. Indeed, there is an increasing body of evidence that suggests that referred sensations, which can include painful and nonpainful reports, are, at least partially, an epiphenomena of the muscles in response to nociceptive stimuli and are not necessarily associated with pathophysiological alterations^2,37,38^. The single report of referred sensation following lidocaine is in line with such previous findings, even though this result lacks statistical significance. It is most likely that the study is underpowered in this regard due to the overall low prevalence of referred sensations in healthy participants^38^. Still, these secondary findings are useful as hypotheses generators.

Overall our findings suggest that peripheral mechanisms seem relevant and might be important contributors of the observed NGF-induced local muscle hyperalgesia. But, it has to be acknowledged that central mechanisms are likely important, although one should keep in mind that central sensitization is an umbrella term that encompasses different mechanisms that are not readily assessed in humans^39,40^. In fact, a complex interaction between peripheral and central mechanisms is responsible for the clinical mosaic of pain sensitivity in musculoskeletal pain disorders, which includes pain-related TMD^41,42^. That being said, the lack of variables that could substantiate that central sensitization is involved, besides the evaluation of the non-injected side, e.g., neurophysiological reflex, secondary hyperalgesia, cutaneous sensitivity and endogenous pain modulation assessment, are considerable limitations of this study. Moreover, we did not assess the success of the double-blind design, although a reasonable likelihood of unblinding can be assumed due to the possible perceptual distortion and other sensory side effects of lidocaine. For instance, successful blinding rates were 15% for participants and 8% for the examiner in an earlier investigation with lidocaine patches^43^. Likewise, blinding was also compromised in the IS group, although the primary comparison was between the NGF + lidocaine and NGF + IS group. Therefore, risk of bias due to unblinding should be taken into consideration. The development of the so-called active placebos^44^ that could cause only the side effects of the lidocaine, e.g., skin reactions or numbness, can be applied to reduce assessment bias in future studies. Finally, further investigations may explore the temporal profile of the analgesic effects in more detail.

In conclusion, local anaesthesia can be successfully applied to reduce the delayed local hyperalgesia present in NGF-sensitized muscles. The clinical implication and novel aspect is that intramuscular injections of local anaesthetics can provide relevant information regarding the contribution of peripheral mechanisms in the maintenance of persistent musculoskeletal pain conditions.

## Methods

### Participants

Forty-five healthy participants were primarily recruited through convenience sampling method from the community of students and staff members of Aarhus University, but also from the general community of Aarhus, Denmark. The following eligibility criteria were applied: (a) age > 18 years and good systemic health with no orofacial and headache pain complaints in the last 30 days or chronic pain disorders (TMD-pain screener were administered to rule out pain-related TMD symptoms)^45,46^; (b) absence of dental or serious medical illness and regular or current intake of antidepressants, anticonvulsants or nonsteroidal anti-inflammatories; (c) any diagnosis of psychiatric or personality disorders. A detailed medical interview was performed to assess the above inclusion and exclusion criteria.

The study was conducted in accordance with the Helsinki Declaration II and after the approval from the Regional Ethics Committee as well as the Danish Data Protection Agency. All participants gave their deliberate and informed consent after a full explanation of all procedures.

### Experimental design

The participants of this randomized, double blind and placebo controlled study were first randomly assigned by a computer-generated list into two groups according to the type of injection that was applied into the right masseter considering a 2:1 allocation ratio: experimental muscle sensitization group (NGF group; n = 30) where NFG 5 µg diluted in 0.2 mL of isotonic saline were injected after a negative aspiration test; control injection group, where the participants received an injection of 0.2 mL of sterile solution of isotonic saline (IS group; n = 15) after a negative aspiration test. In addition, all the participants that received NGF injections were further randomly assigned by a computer-generated list into two equal groups (1:1 allocation ratio) according to the type of injection that was applied into the right masseter 48 h after the NGF injection: intramuscular anaesthetic block (NGF + lidocaine group) where 2 mL of lidocaine 0.5% were injected after a negative aspiration test; (b) intramuscular placebo block (NGF + IS group), where the participants received an injection of 2 mL of sterile solution of IS after a negative aspiration test. The needle was inserted into the centre region of the masseter body perpendicularly to the direction of the muscle fibres until the bone contact was felt, after which the needle was retracted approximately 2 mm, aspiration was done, and the bolus injected in approximately 10 s^47^. A staff member that was not involved in the eligibility evaluation or outcome assessments was in charge of the preparation, randomization and allocation of the injections. Therefore, both the examiner and participants were blinded to the experimental conditions.

**Figure 4:**
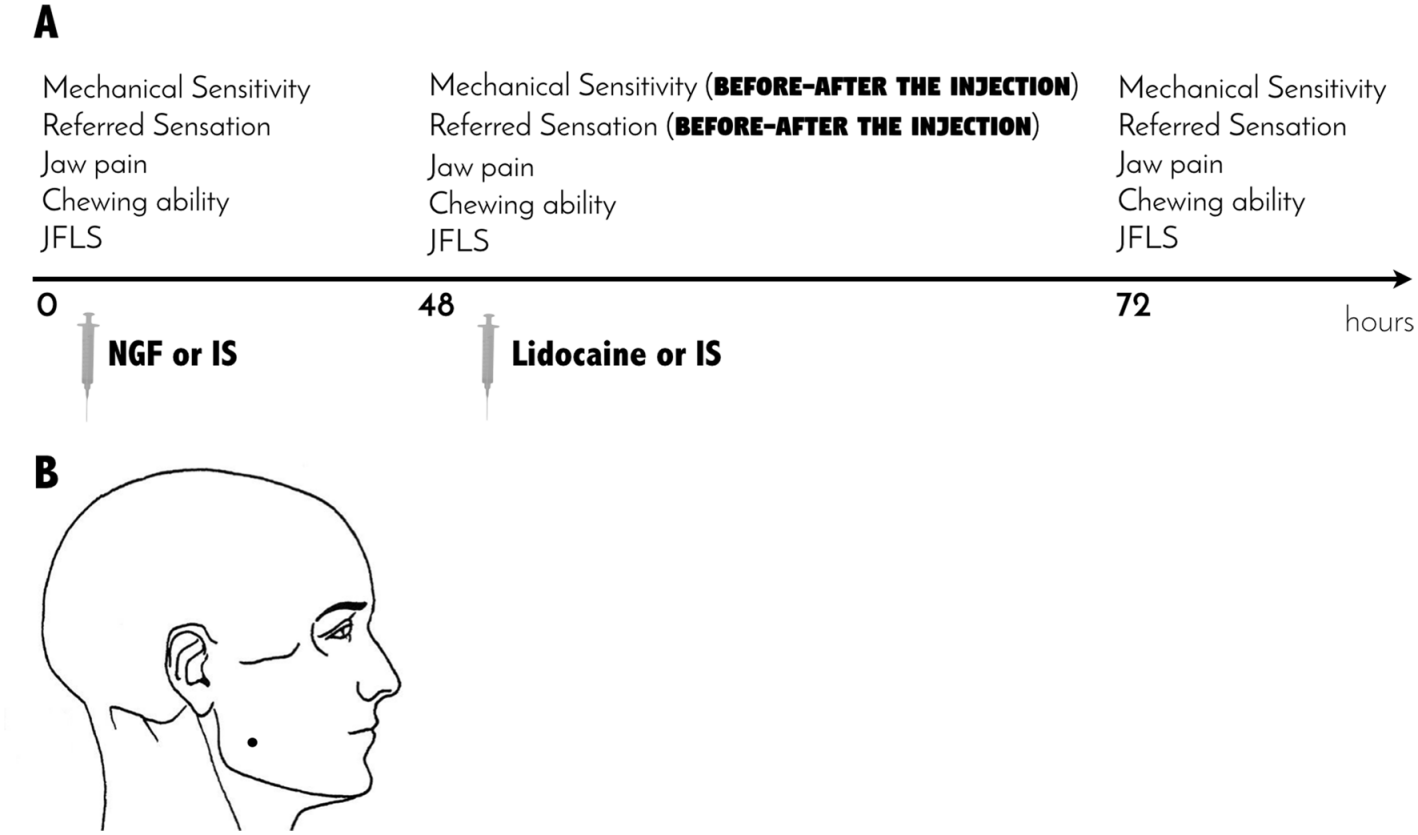
(**A**) Study timeline and outcome variables, which were measured at three or four time points depending on the group and type of variable. Three time points, i.e., baseline (before the injection), 48 and 72 h after the injection, were considered for the group which received isotonic saline (IS; n = 15) or for the clinical assessment of jaw pain intensity and jaw function. On the other hand, four time points, i.e., baseline (before the injection), 48 h after the first injection, 5 min after the second injection of either lidocaine or IS and 72 h after the first injection were considered for the mechanical sensitivity and referred sensations assessments in the groups which received nerve growth factor (NGF + lidocaine; n = 15 and NGF + IS; n = 15). JFLS = jaw functional limitation scale. (**B**) Graphical representation of the injection site (black dot).

The outcome variables were measured at three or four time points depending on the group and type of variable (Fig. 4). Three time points, i.e., baseline (before the injection), 48 and 72 h after the injection, were considered for the IS group or for the clinical assessment of jaw pain intensity and jaw function. On the other hand, four time points, i.e., baseline (before the injection), 48 h after the first injection, 5 min after the second injection of either lidocaine or IS and 72 h after the first injection were considered for the mechanical sensitivity, entropy and referred sensations in the NGF groups (Fig. 4).

### Outcome variables

The primary outcome was the mechanical sensitivity of right (injected side) and left masseter (control side). In addition, the following secondary variables were assessed: entropy of mechanical sensitivity of right and left masseter; presence of referred sensations to pressure stimuli applied to the right and left masseter muscle; clinical assessment of jaw pain intensity and jaw function.

### Mechanical sensitivity and entropy

A complete description of the technique to assess the mechanical sensitivity to pressure stimuli and to calculate the entropy can be found elsewhere^35^. In brief, the area of the right and left masseter was divided into 9 sites and the participant scored the perceived intensity of pressure sensation/pain (0–50–100, numeric rating scale—NRS) of 1 kg and 2 kg force (PALPETER, Sunstar Suisse SA) that was applied on each point for 5 s. The assessment was done with the muscles relaxed and the order of forces, sides and sites was randomized by a computer-generated random list for each participant. Plastic templates applied for each side and in all participants were used to ensure reproducibility of the 9 sites demarcation between the sessions.

The average of the NRS scores from each of the 9 sites was considered as the mechanical sensitivity for that particular side (right and left) and force (1 and 2 kg). Furthermore, each site score was used as a grid value, thus, centre of gravity (CoG) calculation was established, i.e., a representational map of the “centre” of the NRS scores in terms of X and Y coordinates^35^. In the context of diversity of the scores of the masseter muscle, entropy of mechanical sensitivity indicates the degree of diversity of the 0–50–100 NRS scores, with higher entropy values corresponding to more diverse intensity registers of scores over the grid^35^.

### Referred Sensation

The participants were also asked about the experience of referred sensation following the palpation on each of the 9 sites with either 1 or 2 kg force. Thus, the participants were told to let the examiner know if they had experienced sensation that was not only under the tip of the pressure device. If yes, they were asked to point to which regions they had felt the sensation, and the examiner assessed if the region was indeed outside the border of the masseter muscle and counted as a referred sensation site^2,37,38^.

### Jaw pain and function

The intensity of jaw pain at rest and jaw pain evoked by chewing, considering the time frame “in the last 24 h”, was rated on a 0–10 numeric rating scale (NRS), where 0 means “no pain at all” and 10 means “the worst pain imaginable”.

The perceived chewing ability was assessed by the application of three questions from the oral health component of the National Diet and Nutrition Survey from UK, where the answers were scored and summed using a 7-point Likert scale and which indicated low perceived chewing ability in cases of high scores^48^. In addition, we also applied the self-report questionnaire jaw function limitation scale (JFLS-20)^49^. The global score was computed, and higher scores indicate increased overall functional limitation^49^.

### Statistics

It was expected that a medium effect size *f* of 0.25 for the differences in the mechanical sensitivity of masseter would be worth detecting considering the within-between interactions from analysis of variance (ANOVA) with a power of 80%, a significance level of 5% and an anticipated drop-out rate of 25%. Therefore, the sample size estimation was at least 15 participants per group.

The outcome variables were reported as means and standard deviation (SD), unless otherwise noticed. Normal distribution was assessed with the aid of Kolmogorov–Smirnov test and log_10_ transformations were applied for the continuous variables when the results were significant, considering an alpha level of 5% (*p* < 0.050). Thus, the following outcomes were log_10_ transformed: mechanical sensitivity of the control side (left masseter), entropy of mechanical sensitivity of right and left masseter, the intensity of jaw pain at rest and jaw pain evoked by chewing, the perceived chewing ability and jaw functional limitation scale. Normality was re-assessed with the aid of Q-Q plots and the transformed variables were all considered normally distributed.

Mixed design ANOVA was computed to assess differences in the mechanical sensitivity and entropy considering one between-subject factor, i.e., group—3 levels (IS, NGF + lidocaine and NGF + IS), and two within-subject factors, i.e., session—4 levels (baseline, 48 h after the first injection, 5 min after the second injection and 72 h after the first injection) and force—2 levels (1 and 2 kg). In order to allow the running of a single ANOVA model for the primary outcome, the values at “48 h after the first injection” were carried forward to “5 min after the second injection” in the IS group. Pairwise post-hoc comparison analyses were performed using Tukey Honestly Statistical Difference (HSD). The significance level was set at 5% (*p* = 0.050).

Cochran Q and χ^2^ test were applied to compare, respectively, within and between-group differences in the occurrence of referred sensation. A priori Bonferroni correction was applied to adjust the *p* value due to multiple comparisons. Therefore, the significance level for the Cochran Q and χ^2^ test was set at, respectively, 1.6% (*p* = 0.016) and 1.2% (*p* = 0.0125). Due to the limited occurrence of referred sensation, no comparison between forces was made.

Finally, repeated-measures ANOVA was computed to assess differences in the jaw pain at rest and evoked by chewing considering one between-subject factor, i.e., group—3 levels (IS, NGF + lidocaine and NGF + IS) and one within-subject factor, i.e., session—3 levels (baseline, 48 and 72 h after the first injection). Pairwise post-hoc comparison analyses were performed using Tukey Honestly Statistical Difference (HSD). The significance level was set at 5% (*p* = 0.050). In addition, Kruskal Wallis and Friedman test were applied to compare, respectively, between and within-group differences in the self-reported chewing ability and JFLS scores. Pairwise post-hoc comparison analyses were performed using Dunn’s test. The significance level was set at 5% (*p* = 0.050).

## Data Availability

The datasets generated during and/or analysed during the current study are available from the corresponding author on reasonable request.
